# The significance of biomarkers of inflammation in predicting the activity of Lupus nephritis

**DOI:** 10.5937/jomb0-43457

**Published:** 2024-01-25

**Authors:** Violeta Rabrenović, Milica Petrović, Milorad Rabrenović, Dejan Pilčević, Nemanja Rančić

**Affiliations:** 1 Military Medical Academy, Clinic of Nephrology, Belgrade; 2 University of Defense, Military Medical Academy, Faculty of Medicine, Belgrade; 3 Military Medical Academy, Center for Hyperbaric Medicine, Belgrade; 4 Military Medical Academy, Centre for Clinical Pharmacology, Belgrade

**Keywords:** lupus nephritis, activity, NLR, PLR, SIRI, SII, lupus nefritis, aktivnost, NLR, PLR, SIRI, SII

## Abstract

**Background:**

Lupus nephritis (LN) is one of the most severe manifestations of systemic lupus erythematosus (SLE). There are increased studies examining the role of different markers that would facilitate diagnosis, LN activity monitoring, relapse occurrence, and the right time to introduce maintenance therapy. We aimed to examine the importance of determining the neutrophil/lymphocyte ratio (NLR), platelet/lymphocyte ratio (PLR), systemic immuneinflammatory index (SII) and systemic inflammatory response index (SIRI) in LN, comparing their significance with other standard parameters of active disease.

**Methods:**

The clinical examination included 66 patients (34 with active and 32 with LN in remission) and 23 healthy controls. The investigated parameters were CRP, CBC, creatinine, albumin, GFR, C3, C4, ANA, anti-ds DNA Ab, in urine: sediment analysis, SLEDAI/r, proteinuria 24h and Up/cre. We determined the derived markers: NLR, PLR, SIRI, and SII and their correlation with other parameters of active disease.

## Abstract (sr)

**Uvod:**

Lupus nefritis (LN) je jedna od najozbiljnijih manifestacija sistemskog eritemskog lupusa (SLE). Sve je više istraživanja koja ispituju ulogu različitih markera koji bi olakšali dijagnostiku, praćenje aktivnosti LN, pojavu recidiva, kao i pravi trenutak za uvođenje terapije u cilju održavanja remisije. Naš cilj je bio da se ispita značaj određivanja odnosa neutrofila/limfocita (NLR), odnosa trombocita/limfocita (PLR), sistemskog indeksa inflamatornog imunološkog odgovora (SII) i sistemskog indeksa inflamatornog odgovora (SIRI) u LN-u, upoređujući njihov značaj sa drugim standardnim parametrima aktivne bolesti.

**Metode:**

Kliničko ispitivanje je obuhvatilo 66 pacijenata (34 sa aktivnim i 32 sa LN u remisiji) i 23 zdrava kontrolna subjekta. Istraživani parametri su bili CRP, CBC, kreatinin, albumin, GFR, C3, C4, ANA, anti-ds DNK At, a u urinu: analiza sedimenta, SLEDAI/r, proteinurija 24h i Up/cre. Odredili smo izvedene markere: NLR, PLR, SIRI, SII i njihovu korelaciju sa drugim parametrima aktivne bolesti.

**Rezultati:**

Upoređujući grupu sa aktivnim LN-om sa grupom LN-a u remisiji i kontrolnom grupom, dobijena je statistički značajna razlika za CRP (p=0,004), kao i za RBW, hemoglobin, albumin, C3, ANA i anti-ds DNK At (p<0,001), kao i za urinarne parametre, SLEDAI/r, proteinuriju 24h i odnos Up/cre. Upoređujući markere NLR, PLR, SIRI, SII između grupa, može se primetiti značajna razlika za sve odabrane parametre, pri čemu je NLR bio najizraženiji (p<0,001). U aktivnom LN-u, NLR je bio povezan sa CRP-om, kreatininom, SLEDAI/r i proteinurijom 24 h, PLR sa ANA-om, SIRI sa CRP-om, kreatininom, GFR, C3, i anti-ds DNK At-om i SII sa CRP-om. NLR je u zajedničkoj grupi LN, bio parametar sa najvećim značajem u korelaciji sa: C3, albuminom, SLEDAI/r i proteinurijom (p=0,000), ANA-om (p=0,001), anti-ds DNK At (p=0,004) i odnosom Up/cre (p=0,018).

**Zaključak:**

Naši rezultati ukazuju da su NLR, PLR, SIRI i SII povišeni u grupi pacijenata sa aktivnim LN-om i da korelacije ovih biomarkera sa drugim parametrima aktivnosti mogu da budu značajne za procenu bubrežnog oštećenja u LN-a.

## Introduction

Lupus nephritis (LN) represents a lesion of the kidneys, and it is one of the most severe manifestations arising from the progression of systemic lupus erythematosus (SLE), although sometimes it can also be the initial manifestation of SLE. Timely diagnosis and treatment of LN allow not only the recovery of kidney function but also prevent life-threatening complications. This is also the reason for many studies examining the role of various markers that would enable the diagnosis and monitoring of LN activity, the occurrence of potential relapses, and the right time to reduce the dose of immunosuppressive therapy [1] [2] [3] [4] [5]. Potential markers whose determination is not complicated, which do not require a long process, and are available in everyday clinical practice are significant. In recent years, several studies have been published which showed the importance of blood-derived markers such as neutrophil/lymphocyte ratio (NLR), platelet/lymphocyte ratio (PLR), systemic immune-inflammatory index (SII) and systemic inflammatory response index (SIRI) in the prediction of various diseases [6] [7] [8] [9] [10] [11] [12]. Neutrophils, lymphocytes and platelets play a significant role in the inflammatory response, which is a factor in the progression of many autoimmune diseases [13] [14]. These haematological parameters have a predictive value that increases when we express them through ratios or indices [14] [15]. NLR is a marker of neutrophilic inflammation, potential infection and organ lesions, and PLR is an indicator of inflammatory and prothrombotic states of many diseases (malignancies, rheumatic diseases, cardiovascular diseases [16].

SII is a combination of three haematological parameters (platelets, neutrophils, and lymphocytes) and its elevated values are described as an unfavourable prognostic parameter in many conditions(surgical diseases, neoplasia, coronary artery disease, in the progression of atherogenesis, in antineutrophil cytoplasmic antibody-associated-ANCA vasculitis, etc.) [12] [15] [17] [18]. SIRI is an index derived from a combination of neutrophils, lymphocytes and monocytes and is related to the status of immune defence. It indicates the repair processes of damaged tissues after inflammation and an immune response that can damage tissues, so it represents a prognostic parameter for many postoperative conditions. However, few studies examine these markers’ significance in SLE, especially in LN. We aimed to examine the significance of the determination of NLR, PLR, SIRI and SII in LN, comparing their significance with other standard parameters of LN activity.

## Materials and methods

In the clinical examination (approved by the Ethics Committee and performed according to thetenets of the Declaration of Helsinki and conducted from 2012–2019), we included a group of 66patients and 23 healthy controlsof both sexes, older than 18 years, who were examined and treated in the Clinic of nephrology, Military Medical Academy, Belgrade. In a patient with SLE, the diagnosis was confirmed by the criteria of the American College of Rheumatology (ACR) and European League Against Rheumatism (EULAR) [19] [20]. LN was confirmed by kidney biopsy and pathohistological verification (WHO classification, and the International Society of Nephrology/Renal Pathology Society (ISN/RPS) classification [21].

Kidney disease activity was also classified according to the renal disease activity index SLEDAI/r (Systemic Lupus Erythematosus Disease Activity Index/renal [22]. SLEDAI/r consists of 4 criteria that grade renal impairment within the SLEDAI 2000 (Systemic Lupus Erythematosus Disease Activity Index- SLEDAI 2000) criteria of SLE activity [22].The patients were divided into three groups: the first group consisted of patients with LN who had active disease (34–38.2%), the second group consisted of patients with LN in remission (32–35.9%), and the third group – the healthy control group (23 - 25.8%).The first group who had the active disease, which according to standard analysis was defined as proteinuria 0.5 g/24 h: urinary protein/creatinine ratio > 0.5: according to SLEDAI/r criteria (> 4), hypocomplementemia C3, C4, positive anti-double stranded DNA antibodies (anti-ds DNA Ab) and pathohistological findings of renal biopsy. All patients had a glomerular filtration rate (GFR) of 60 mL/min/1.73 m^2^, according to the Chronic Kidney Disease Epidemiology Collaboration (CKD-EPI) [23].The second group: consisted of patients with SLE and LN who were in complete remission (according to the criterion: proteinuria 0.5 g/24h., urinary protein/creatinine ratio <0.5: SLEDAI/r criteria (<4) negative anti-ds DNA antibodies, complement C3 and C4 within the reference range, and GFR 60 mL/min/1.73 m^2^).The third group – the healthy control group – consisted of patients who did not have SLE and LN: that is, they did not have autoimmune diseases. It is characteristic for them that the kidney function was preserved (GFR 60 mL/min /1.73 m^2^). Excluding criteria were the same for all groups: patients with infection, positive urine culture, with kidney failure (CKDeGFR<60 mL/min/1.73 m^2^). Also excluding criteria were other autoimmune diseases, inflammatory diseases, malignant diseases, and haematological diseases, as well as patients who were on corticosteroid therapy for some other reasons, that is, they had repeated transfusions. All laboratory parameters for the first group were determined before the immunosuppressive treatment started (in this way, the effect of the therapy on the laboratory analysis was prevented). The second group included patients in remission, which was maintained with 5–10 mg of corticosteroids and Azathioprine 50–75 mg per day. The patients in the third group did not receive immunosuppressive therapy.The authors had access to information which identified participants in the study.

We monitored standard laboratory and kidney function parameters: C reactive protein (CRP), Complete Blood Count, creatinine, albumin, and GFR. Regarding immune parameters, complement C3 and C4, antinuclear antibodies (ANA), and anti-ds DNA Ab were monitored. Urine sediment, SLEDAI/r, proteinuria 24h, urine culture, and the ratio of urinary proteins and creatinine (U p/cre) were monitored in urine. We also determined the derived markers: NLR, PLR, SIRI, and SII.

NLR was calculated by dividing the absolute neutrophil count (ANC) by the absolute lymphocyte count (ALC). In contrast, PLR was calculated by dividing the absolute platelet count (PLT) by the ALC. We also determined the SII – which indicates the quotient of the product of platelets and neutrophils with lymphocytes (P x N)/L, where P, N, and L represent the peripheral platelet, neutrophil, and lymphocyte counts, respectively. SIRI – the response index to systemic inflammation, is defined as (N x M)/L, where N, M, and L represent the counts of peripheral neutrophils, monocyte, and lymphocyte. The markers were calculated as follows:

NLR = Neutrophil count (10^9^/L)/ Lymphocyte count (10^9^/L):

PLR = Platelet count (10^9^/L)/ Lymphocyte count (10^9^/L):

SIRI = (Neutrophil count (10^9^/L) × Monocyte count (10^9^/L))/ Lymphocyte count (10^9^/L):

SII = (Platelet count (10^9^/L) × Neutrophil count (10^9^/L))/ Lymphocyte count (10^9^/L).

### Statistical analysis

The data were analysed using the Statistical Package for the Social Sciences IBM-SPSS, version 26.0. Categorical variables were presented as frequency and were analysed using the Chi-square test. All continuous variables are presented as median (interquartile range: 25–75th percentile) or mean±standard deviation for the data that are not normally or normally distributed, respectively. The Kolmogorov-Smirnov test was used to test the normality of data distribution. The Kruskal-Wallis test or Mann-Whitney test for non-parametric variables was used for intergroup comparisons. Spearmen’s coefficient correlation tested the relationship between variables. Optimal thresholds (cut-off) of biomarker values (SIRI, SII, PLR, and NLR) for assessment of LN activity were determined by receiver operating characteristic (ROC) curve analysis. Statistical significance was defined as p<0.05 for all comparisons. Significance of differences was accepted at three levels of significance: * <0.05: ** < 0.01: *** <0.001.

## Results

In the total group of respondents (n=89), the female gender dominated in the ratio of 71.9%: 28.1% to the male gender. The average age of our subjects was 45.97±14.35 years, whereby the group with active LN was 40.76±16.51 years old, the group with LN in remission was 45.09±11.65 years old, and the control group was 54.87±9.89 years old. The basic laboratory analysis for all three groups is shown in Table 1.

**Table 1:** Comparison between Patient Groups with LN and control group regarding laboratory data.

Parameters (IQR)	LN active group<br>N=34	LN remission group<br>N=32	Control group<br>N=23	p-value
CRP (mg/L)	3.47 (2.58–5.50)	3.42 (3.10–4.10)	2.09 (1.20–2.93)	**0.004**
WBC (10^9^/L)	5.54 (4.84–7.06)	6.71 (4.55–8.12)	6.36 (5.06–6.90)	0.513
RBC (10^9^/L)	3.89 (3.46–4.28)	4.33 (4.01–4.71)	4.77 (4.63–5.23)	**<0.001**
Hb (mg/L)	111.00 (92.00–120.00)	122.50 (116.25–132.00)	141.00 (134.00–146.00)	**<0.001**
PLT (10^3^/μL)	195.50 (171.50–243.75)	212.50 (180.00–268.75)	211.00 (194.00–233.00)	0.424
CREATININE (μmol/L)	80.50 (64.00–130.25)	78.50 (67.00–106.50)	72.00 (60.00–77.00)	**0.023**
GFR (mL/min/1,73m^2^)	81.00 (58.49–108.00)	78.27 (65.81–97.50)	96.80 (93.50–98.96)	**0.012**
ALBUMIN (g/L)	34.00 (27.00–37.25)	40.00 (38.00–42.75)	44.00 (43.00–45.00)	**<0.001**
C3 (g/L)	0.72 (0.50–6.18)	0.84 (0.81–1.01)	/	**<0.001**
C4 (g/L)	0.10 (0.05–0.14)	0.14 (0.12–0.18)	/	**0.002**
ANA (IU/mL)	3.00 (2.00–3.00)	0.00 (0.00–1.00)	/	**<0.001**
Anti-ds DNA Ab (IU/mL)	90.00 (47.50–137.00)	15.00 (15.00–15.00)	/	**<0.001**
Proteinuria g/24h	3.55 (1.86–4.59)	0.26 (0.16–0.37)	0.15 (0.07–0.27)	**<0.001**
Up/cre	2.10 (0.93–2.39)	0.23 (0.18–0.26)	/	**<0.001**
SLEDAI/r	6.00 (3.75–7.00)	0.00 (0.00–1.00)	s/	**<0.001**

Data are expressed as Median (IQR). Kruskal Wallis Test or Mann-Whitney test were used (bold values are significant). C reactive protein, CRP: white blood cell count, WBC: red blood cell count, RBC: hemoglobin, Hb: platelet count, PLT: glomerular filtration rate, GFR: complement C3, C3: complement C4, C4: antinuclear antibodies, ANA: anti-double stranded DNA antibodies, anti-ds DNA Ab: 24 hour urinary proteins, proteinuria g/24h, urinary proteins and creatinine ratio, U p/cre: renal disease activity index, SLEDAI/r.

Comparing the group with active LN, the group with LN in remission and the control group, a statistically significant difference was obtained for the parameter of non-specific inflammation – CRP (p=0.004), and analysing the complete blood count, a statistically significant difference was observed for RBW and hemoglobin (p<0.001) which were the lowest in the group with active LN, while the difference for PLT and WBC was not significant. In the biochemical analyses, statistical significance is observed in the difference in parameters related to disease activity: albumin and complement C3 (p<0.001) were the lowest in the group with active LN, with slightly lower significance for C4 (p=0.002) between the two groups with LN, and for ANA and anti-ds DNA Ab, the expected statistically significant difference was obtained (p<0.001). Analysing urinary parameters, statistical significance was obtained for SLEDAI/r index, proteinuria 24h and Up/cre ratio, which were elevated in the group with active LN.

Data are expressed as *Median (IQR)*. Kruskal Wallis Test was used (bold values are significant). Neutrophil/lymphocyte ratio, NLR: Platelet/lymphocyte ratio, PLR: System inflammation response index, SIRI: systemic immune inflammation index, SII.

**Figure 1:**
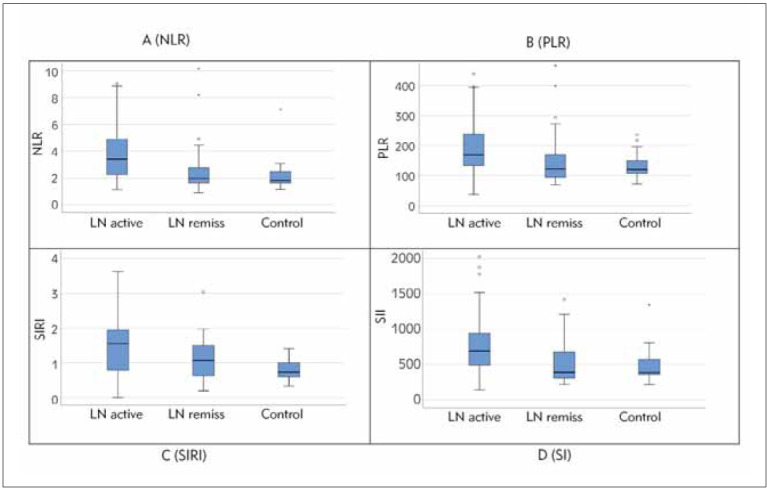
Comparison of levels of neutrophil/lymphocyte ratio (A), platelet/lymphocyte ratio (B), systemic immune inflammation index (C) and systemic inflammation response index (D) among the patients with LN active form, LN in remission and control group.

Comparing the difference in leukocyte subgroups in Table 2, significance was obtained only forlymphocytes with the lowest level in the group with active LN. In Table 2, as well as in the graphic representation (Figure 1), we have shown comparisons of the parameters NLR, PLR, SIRI, and SII, between the groups, and statistically significant differences were obtained for all selected parameters, with NLR being the most significant (p<0.001).

**Table 2:** between LN patients with activity, LN patients in remission and control group as regard NLR, PLR, SIRI, SII.

	LN active group	LN remission group	Control group	p-value
Neutrophil (10^9^/L)	4.10 (3.10–5.04)	3.94 (2.95–5.08)	3.40 (2.80–4.30)	0.213
Lymphocyte (10^9^/L)	1.20 (0.83–1.50)	1.80 (1.24–2.15)	1.75 (1.46–1.99)	**0.002**
Monocytes (10^9^/L)	0.32 (0.20–0.60)	0.50 (0.30–0.67)	0.38 (0.29–0.53)	0.080
NLR	3.39 (2.23–5.18)	1.97 (1.64–2.86)	1.83 (1.64–2.52)	**<0.001**
PLR	168.52 (132.51–257.08)	121.89 (91.90–169.32)	119.41 (106.91–155.31)	**0.011**
SIRI	1.55 (0.75–1.95)	1.06 (0.62–1.51)	0.73 (0.59–1.01)	**0.005**
SII	692.00 (457.99–991.94)	390.53 (307.30–704.55)	387.96 (356.40–571.38)	**0.004**

If we look at the group with active LN in Table 3, we notice that there is a statistically significant correlation for NLR with CRP, with parameters of renal function (creatinine) as well as with SLEDAI/r and proteinuria 24h. PLR correlates statistically significantly with ANA. The SIRI parameter had a significant correlation with CRP, creatinine and GFR, as well as with the immune parameters C3, and anti-ds DNA Ab. The SII parameter statistically correlated with CRP. According to these correlations, in the active LN group, two parameters, NLR and SIRI, can be distinguished, which showed the greatest statistical significance.

**Table 3:** Correlation of selected markers (NLR, PLR, SIRI, SII) in the group with active LN with standard parameters for LN activity. Spearman’s correlation rank (bold values are significant)

Parameter<br>x	Statistical<br>parametes	Parameter y									
		CRP	Creatinin	GFR	Albumin	C3	ANA	Anti-ds<br>DNA Ab	SLEDAI/r	Protein/<br>u 24h	Up/cre
**NLR**	Coeff.correlation<br>(r)	**-0,921**	**- 0,793**	0.621	-0.210	-0.098	0.766	0.142	**0.794**	**-0.923**	-0.611
	Probability	**0.018**	**0.047**	0.088	0.221	0.288	0.053	0.261	**0.046**	**0.017**	0.115
**PLR**	Coeff.correlation<br>(r)	-0.107	- 0.753	0.738	-0.513	-0.088	**-0.976**	0.211	-0.534	-0.759	-0.388
	Probability	0,548	0.056	0.059	0.116	0.297	**0.005**	0.224	0.111	0.055	0.194
**SIRI**	Coeff.correlation<br>(r)	**-0.892**	**0.896**	**-0.968**	0.698	**0.956**	0.773	**0.959**	-0.067	-0.545	-0.320
	Probability	**0.024**	**0.024**	**0.007**	0.070	**0.010**	0.052	**0.009**	0.323	0.109	0.228
**SII**	Coeff.correlation<br>(r)	**0.825**	-0.668	0.464	-0.535	-0.199	-0.744	0.308	-0.393	-0.437	-0.278
	Probability	**0.040**	0.077	0.132	0.112	0.229	0.059	0.186	0.154	0.140	0.242S

In the group of patients with LN, which were observed collectively (patients with an active form andin remission – a total of 66 patients), we noticed that the correlation between NLR, PLR, SIRI, and SII with CRP, creatinine, GFR is statistically insignificant. We observed that NLR in these patients was the most significant parameter in correlations with albumin, C3, SLEDAI/r index, and proteinuria 24h, the significance was p=0.000, and for ANA (p=0.001), antids DNA Ab (p=0.004), Up/cre (p=0.018) was slightly smaller. Among other parameters, PLR significantly correlated with C3 (p=0.000), ANA (p=0.014), SLEDAI/r index (p=0.033) and proteinuria (p=0.040). SII showed a significant correlation with the same parameters C3 (p=0.027), ANA (p=0.020), anti-ds DNA Ab (p=0,019), SLEDAI/r index (p=0.008) and proteinuria 24h (p=0.003), but also with albumins (p=0.002). Statistical significance in the correlations related to SIRI with selected parameters in the combined LN group was the lowest: It was observed only for albumin (p=0.025) and proteinuria 24h (p=0.027), and the other correlations were not significant. Table 4

**Table 4:** Correlation of selected markers with standard parameters for LN activity in the collective group LN (the group with active LN and group with LN in remission). Spearman’s correlation rank (bold values are significant).

Parameter x	Statistical<br>parameters	Parameter y									
		CRP	Creatinin	GFR	Albumin	C3	ANA	Anti-ds<br>DNA	SLEDAI/r	Protein/<br>u 24h	Up/cre
NLR	Coeff. correlation (r)	-0.001	0.094	-0.043	**-0.454**	**-0.438**	**0.416**	**0.352**	**0.417**	**0.452**	**0.358**
	Probability	0.995	0.453	0.734	**0.000**	**0.000**	**0.001**	**0.004**	**0.000**	**0.000**	**0.018**
PLR	Coeff. correlation (r)	-0.098	-0.057	0.055	-0.233	**-0.432**	**-0.301**	0.217	**0.263**	**0.254**	0.113
	Probability	0.432	0.651	0.656	0.060	**0.000**	**0.014**	0.085	**0.033**	**0.040**	0.469
SIRI	Coeff. correlation (r)	0.027	0.084	-0.081	**-0.278**	-0.226	0.207	0.137	0.200	**0.275**	0.203
	Probability	0.832	0.505	0.521	**0.025**	0.070	0.098	0.286	0.109	**0.027**	0.198
SII	Coeff. correlation (r)	0.065	0.011	0.019	**-0.380**	**-0.274**	**0.287**	**0.052**	**0.327**	**0.367**	0.232
	Probability	0.610	0.932	0.878	**0.002**	**0.027**	**0.020**	**0.019**	**0.008**	**0.003**	0.134

**Figure 2:**
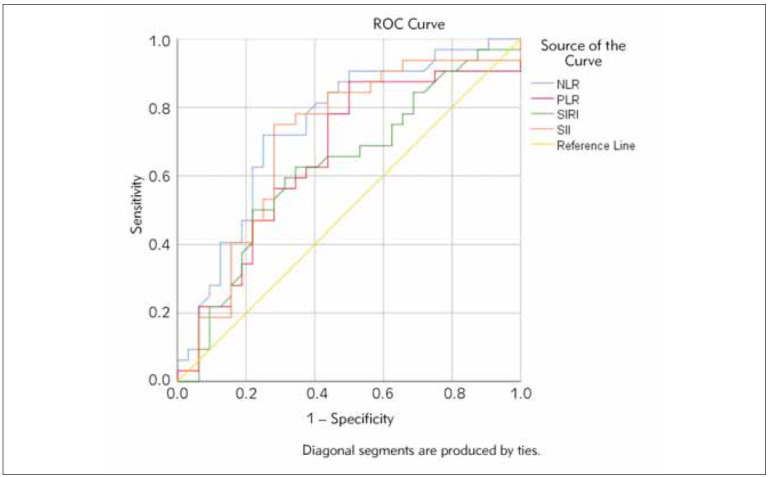
Receiver operating characteristic (ROC) curve of NLR, PLR, SIRI, SII for disease activity in LN.

The ROC analysis of the NLR, PLR, SIRI, and SII are shown in Table 5 and Figure 2. The area under the ROC (AUC) value of the NLR was 0.747, the best cut-off value was 2.670 (p=0.001), the AUC value of the PLR was 0.658, and the best cut-off value was 116.625 (p=0.0810). The AUC value of the SIRI was 0.627, the best cut-off value was 1.225 (p=0.081), the AUC of the SII was 0.708, and the best cut-off was 500.145 (p=0.004).

**Table 5:** Receiver operating characteristic curve analysis of NLR, PLR, SIRI, SII.

Test Result<br>Variable(s)	Area	Asymptotic Sig	Asymptotic 95% CI				
			Lower Bound	Upper Bound	Sensitivity	Specificity	Cut-off value
NLR	0.747	0.001	0.624	0.869	0.719	0.750	**2.670**
PLR	0.658	0.030	0.521	0.796	0.875	0.500	**116.625**
SIRI	0.627	0.081	0.488	0.766	0.625	0.656	**1.225**
SII	0.708	0.004	0.576	0.840	0.750	0.719	**500.145**

## Discussion

SLE is a disease characterized by the impaired of immunological self-tolerance and production ofautoantibodies, creation and deposition of immune complexes, activation of the complement system, and chronic inflammation [24]. This is also why in patients with SLE, we observe elevated parameters of inflammation, such as CRP, ESR, and IL6, the increase of which indicates the progression of the disease [25]. The systemic inflammation that we encounter in SLE and many other diseases is also characterised by an increase in NLR and PLR markers, which also have predictive value, according to previous research [8] [10] [15] [16] [17]. SII and SIRI markers are less described than NLR and PLR, especially in SLE and LN.

In our research, we concluded that there is a statistically significant difference between the groups for the non-specific inflammation parameter CRP and lymphocytes (increased CRP values and lymphopenia) in the group with active LN. At the same time, neutrophils and monocytes did not significantly differ between the groups. This can be explained by the fact that SLE is characterised by leukopenia, a diagnostic criterion and parameter of disease activity.

Our results were similar to the results of Han et al., who concluded that lymphopenia is the most critical element in the assessment of SLE activity [26]. At the same time, neutrophils provide insight into other immunopathological mechanisms [26]. They specifically investigated the association of NLR and SLE activity and concluded that NLR is elevated in active disease, as well as circulating immune complexes and anti-ds DNA Ab [26].

In our group of patients with active LN, there was also an increase in NLR and other parameters of active disease: ANA, anti-ds DNA Ab, SLEDAI/r index, and proteinuria. We supplemented our research with other haematological markers. In addition to NLR, the values of other parameters of PLR, SII and SIRI were increased in patients with LN compared to the control group. When we compared all three groups, a statistically significant difference emerged between patients with active LN, LN in remission, and the control group, which had the highest significance for NLR (p<0.001).

A group of Egyptian researchers described similar results in a study that included 110 patients with SLE (80 with active form of LN and 30 without LN), where NLR and PLR were significantly elevated in SLE (p=0.007), as well as in patients with active LN (p<0.001) [27]. In their study, NLR and PLR significantly correlated with other parameters of active disease: ESR, hypocomplementemia, IL6, proteinuria and Systemic lupus international collaborating clinicsdamage index-SLICC DI index. They concluded that NLR and PLR would be potentially useful and inexpensive markers for SLE AND LN disease activity [27].

What we observed for the correlations of NLR and PLR parameters with standard parameters ofactive disease in the group of patients with LN is the statistically significant correlation of NLR with albumin, C3, ANA, anti-ds DNA Ab, SLEDAI/r score, proteinuria 24h and Up/cre. In our study, the PLR parameter correlated statistically significantly with complement C3, ANA, SLEDAI/r score and proteinuria 24h. We concluded that NLR showed a better correlation for LN activity than PLR. Similar results were described by Suszek et al., who noted that elevated NLR in SLE is an indicator of organ and tissue lesions (kidneys, skin and mucous membranes mainly). That elevated PLR was also observed in haematological involvement and renal lesions [28].

In our study, SII showed a significant correlation with several parameters for LN activity (albumin, C3, ANA, anti-ds DNA Ab, SLEDAI/r score and proteinuria 24h), which is explained by the fact that systemic immune inflammatory is particularly pronounced in active disease. This is also observed when comparing the value of SII in patients with active LN, which is almost twice as high compared to the group with LN in remission and the control group. This is also confirmed in the study by Ozdemir et al., SII is elevated in active SLE, but they emphasise that NLR is a better predictor of activity than SII, especially for the occurrence of nephritis [29]. Data from studies determining SIRI in SLE and LN are very scarce. Many more works indicate its prognostic significance in survival in malignant diseases and immune status in viral infections (Covid-19) [30] [31]. As a parameter of theimmune response and a parameter significant in tissue regeneration processes, SIRI was significantly higher in our group with active LN compared to the other two groups (p=0.005). The correlation of SIRI in our group of patients with active LN was significant with the non-specific inflammation parameter CRP, creatinine GFR, C3, and anti-ds DNA Ab, but not with urinary parameters. At the same time, SII correlated significantly only with CRP. NLR is the only marker that is signifi cantly correlated with with proteinuria 24h and creatinine in this group. Soliman et al.’s study concluded that patients with SLE without nephritis had elevated values of NLR and PLR thatcorrelate with CRP, C4, and SLEDAI index [32]. However, the group with LN had a higher value of NLR than the group without LN, and a significant correlation with creatinine and proteinuria 24h [32]. Similar to our study, their results indicate the importance of NLR and PLR as parameters of renal lesions [32].

Comparing the sensitivity and specificity in our group with LN, we observed that NLR had a specificity of 75%, sensitivity of 71.9%, PLR sensitivity of 87.5% and specificity of 50%. The cut-off value we obtained for NLR was 2,670, similar to the cut-off value of 2,260 (sensitivity 75%, specificity 50%) described by Wu et al. [33] for active SLE in their study. The threshold value described by Abdulrahman et al. [27] for PLT was 316.5, while in our study, it was lower and was 116.62. Ozdemir et al. [29] conclude that SII predicts active SLE (AUC 0.626) and PLR (AUC 0.66). However, they also state that the best prediction of active disease is NLR (AUC 0.723), and the threshold value obtained in their study is 2.32 [29].

## Conclusion

Our results indicate that inflammation markers NLR, PLR, SIRI, and SII are statistically significantly elevated in patients with active lupus nephritis. Correlations of those biomarkers with other standard parameters for LN activity may be significant for evaluating renal lesions in LN.

## Dodatak

### Statement of ethics

The Ethics Committee of our institution approved the study, and oral informed consent was obtained from all patients.

### Informed consent

Informed consent was obtained from all individual participants included in the study.

### Conflict of interest statement

All the authors declare that they have no conflict of interest in this work.
